# Influencing Factors on Health Information to Improve Public Health Literacy in the Official WeChat Account of Guangzhou CDC

**DOI:** 10.3389/fpubh.2021.657082

**Published:** 2021-08-03

**Authors:** Xiaowei Ma, Jianyun Lu, Weisi Liu

**Affiliations:** ^1^Department of Public Health Emergency Preparedness and Response, Guangzhou Center for Disease Control and Prevention, Guangzhou, China; ^2^Department of Infectious Disease Control and Prevention, Guangzhou Center for Disease Control and Prevention, Guangzhou, China; ^3^Department of Health Education and Promotion, Guangzhou Center for Disease Control and Prevention, Guangzhou, China

**Keywords:** WeChat, official account, social media, health information, public health emergency literary

## Abstract

**Background:** Social media is used as a new channel for health information. In China, the official WeChat account is becoming the most popular platform for health information dissemination, which has created a good opportunity for the Centers for Disease Control and Prevention to facilitate health information online to improve emergency public health literacy.

**Methods:** Data were collected from the Guangzhou CDC i-Health official WeChat account between April 1, 2018 and April 30, 2019. Descriptive analysis was performed for basic information about the followers and posts of the official WeChat account. Multiple logistic regression analysis was used to analyze the association among various factors of posts on engagement of followers of the official WeChat account.

**Results:** Among 187,033 followers, the total numbers of post views, shares, likes, add to favorites, and comments for 213 posts were 1,147,308, 8,4671, and 5,535, respectively. Engagement of followers peaked on the dissemination date and gradually declined. The main post topics were health education posts and original posts. In the multiple logistic regression model, the number of post views was found to be significantly associated with infectious disease posts (AOR: 3.20, 95% CI: 1.16–8.81), original posts (AOR: 10.20, 95% CI: 1.17–89.28), and posts with title-reflected content (AOR: 2.93, 95% CI: 1.16–8.81).

**Conclusion:** Our findings facilitate the government to formulate better strategies and improve the effectiveness of public information dissemination.

## Introduction

Social media is becoming the most important channel for the public to obtain health information (1–3). Meanwhile, the government has tended to use social media to improve public health literacy to prevent infectious diseases (4, 5). Social media platforms such as Facebook (6–8), Twitter (9, 10), and Instagram (10, 11) were wildly used for health information. WeChat, a free mobile app released in 2011, is the most popular social media platform in China, and it had 1.04 billion monthly active users worldwide by the first quarter of 2018, and 93% of residents in major Chinese cities are reported to log into WeChat daily (12). WeChat is widely used in many scenes, such as traffic classification (13), health information (12), and so on. A recent national survey in China found that one-third of participants regularly read health information articles on WeChat, and 98.53% of participants chose to use WeChat for health information seeking, indicating that the official WeChat account (OWA) is the most popular platform for health information acquisition in China (12). Due to growing online health-seeking behaviors, increasing numbers of non-authorized social media accounts are sharing biased or inaccurate health information, it is urgent for health organizations to engage with users on social media in a strategic and technological way (14, 15). WeChat, Twitter, and Facebook, the three most popular social media platforms globally, differ substantially in functionality, including text content, images, video, and setting of post objects (1, 10, 11, 16, 17). OWA is an application account supplied by administrators on the WeChat public platform, which can be used for communication and interaction with specific groups via text, pictures, voice, and video. Members of the public can follow OWAs of interest to receive relevant information or messages. The OWA of the CDC is considered as an appropriate and professional platform for informing, educating, and empowering the public regarding health issues (18–20).

At present, a lot of studies about the influencing factors on health information by social media, including Facebook, Twitter, and Instagram, have been reported (14, 21–23). However, there are few such studies about the influencing factor of health information on an OWA (24). Studies focused on the engagement of followers and influence on the OWA of the CDC is sparse. Hence, it is important to explore the influencing factors mentioned above to get a better strategy to disseminate health information. The Guangzhou Center for Disease Control and Prevention i-Health official WeChat account (GZCDC i-Health OWA) opened in April 2018 and is managed by specialized staff at the GZCDC. Posts published via this OWA are mainly original, and focus on health tips and popular science. In our study, we analyzed the data from the posts published between April 2018 and April 2019 to explore the key factors on health information by the GZCDC i-Health OWA to improve the strategies toward health information and promote public health literacy.

## Materials and Methods

### Data Collection and Logging

Data were collected from the GZCDC i-Health OWA which disseminates health information to the public. Gender and regional distribution were indicated in individual profiles. There are three main ways for WeChat users to read the articles. First, users can get health information directly from OWAs they follow. Second, users can receive health information through “Moments,” a functional mode of WeChat by which users can see their friends' posts. Finally, users can read the articles transmitted by friends. According to the operational rules, an administrator can only post once a day, with one or more posts each time. The administrator can also select a post to be the headline post. Two investigators, who received training regarding the purpose of the study and the data collection procedures, used the same standard for classification of the variables throughout the study. We used double logging of data and conducted a consistency check for the collected data.

### Inclusion Criteria

All posts published by the GZCDC i-Health OWA dated from April 1, 2018 to April 30, 2019 (end of data collection) were included in this study.

### Variable and Characteristics

All information was classified into different types of variables. The categorical variables included gender and regional distribution of followers, types and characteristic of posts, effectiveness of dissemination, headline, and title-reflected content. Types of posts were categorized into health education posts and organizational promotion posts. Health information posts include information or news articles on a range of health topics delivered to the public or professionals, while organizational posts cover content or messages designed to advertise or build the image of an organization or publish a notification. In other words, organizational promotion posts do not contain any health content. Based on the content of posts, we divided health information posts into six subcategories, including infectious disease, vaccine-associated event, environment health, nutrition and food-borne disease, child and adolescent health, and chronic disease, then we evaluated the most effective type of post for engaging followers. According to the copyright by the GZCDC, the posts were categorized into original posts and reposts. The position of posts was categorized into headline posts and non-headline posts. The effectiveness of posts was categorized depending on whether posts ran hot spots or not. “Running hot spots” refers to choosing topics that are close to news hot spots, addressing the health content of the audience's current concerns, and conveying the most important viewpoints and accurate information to the audience as quickly as possible (25). Whether the title reflected the content was the last independent variable. If the post content could be judged from the title, it was categorized as “Yes,” and if not, it was categorized as “No.”

In addition, continuous variables included the number of followers and the engagement of followers 7 days after the post was published, including the number of post views, reposts, and adding to favorites for each post. Engagement of followers was defined as the total number of interactive behaviors of followers, including views of each post, amounts of sharing, and adding to favorites.

### Statistical Analysis

Descriptive statistical analysis was performed for the continuous variables. We performed multiple logistic regression, which was utilized in previous studies (14, 21, 26), to explore the influence factors on the engagement of followers of GZCDC i-Health OWA. We transformed a continuous dependent variable into a binary variable, because the data did not follow a normal distribution. For the engagement of followers, the median volume was used as the cut-off point to distinguish “good engagement” (equal to or larger than the median) and “poor engagement” (less than the median). For the type of posts, organizational posts that did not contain health content were set as the reference. For the characteristics of posts, reposts were set as the reference. For the effectiveness, the comment event was chosen as the reference. For the headline and whether the title reflects the content section, non-headlines and titles that did not reflect the content were set as the reference. All independent variables were included with a forced entry method. Adjusted odds ratios (ORs) and corresponding 95% confidence intervals (CIs) for the factors were computed. *P* < 0.05 were defined as statistically significant. All analyses were conducted using R software 3.4.2.

### Ethics Approval

This study was approved by the ethics committee of the Guangzhou CDC. Our study did not involve any private and personal information. All data were anonymous.

## Results

**Figure 1:**
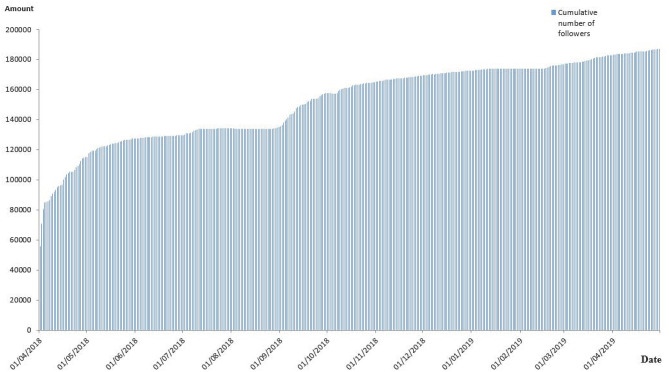
Cumulative number of followers of Guangzhou CDC I-Health WeChat Official Account during Apr. 2018 - Apr. 2019.

The GZCDC i-Health OWA had 187,033 followers in April 2019, compared to 55,702 in April 2018. Among these, 24.94% (*n* = 46,646) were men, and 75.06% (*n* = 140,378) were women. A total of 95.87% of followers (*n* = 182,154) were in Guangdong Province, and 4.13% followers (*n* = 4,898) were located in other provinces and cities. Figure 1 shows the increased cumulative followers of the Guangzhou i-Health official WeChat account from April 2018 to April 2019. The GZCDC WeChat account posted 213 posts from April 2018 to April 2019. Overall, 23.47% of posts were organizational promotion posts, while 76.53% were health information posts, which were classified into six subcategories for further study. In addition, 91.55% of posts were original posts, 87.32% of posts were headline posts, 22.54% of posts were focused on a current event, and 46.48% of posts were reposted by other OWAs.

**Figure 2:**
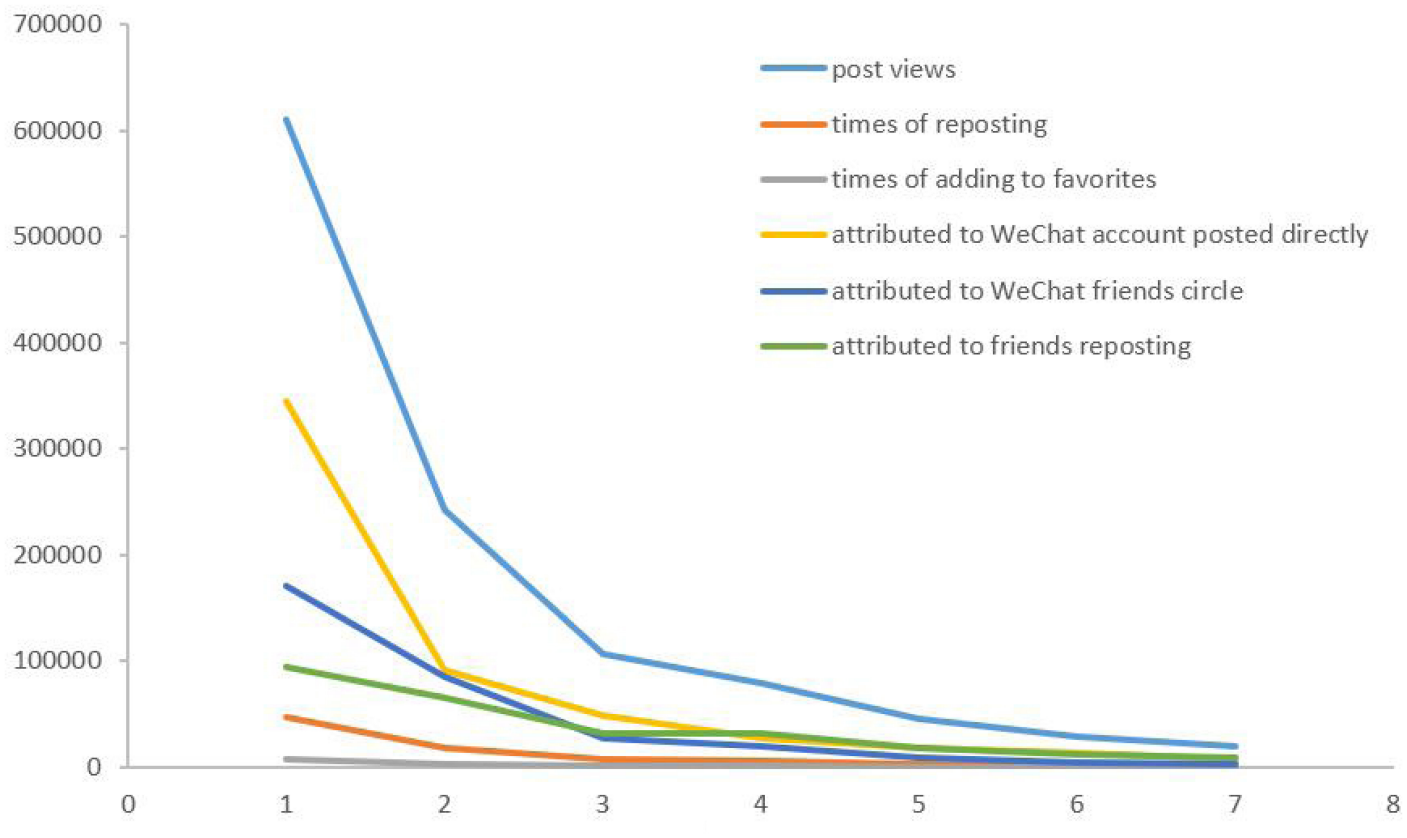
Engagement of followers in the 7 days after the posts posted.

The total numbers of post views, shares, and adds to favorites for 213 posts were 1,147,308, 84,671, and 5,535, respectively. The proportions of direct posts, WeChat friend's circles, and friends sharing were 48.18% (552,804/1,147,308, 95% CI: 48.09%−48.14%), 27.72% (318,003/1,147,308, 95% CI: 27.64%−27.80%), and 24.10% (276,501/1,147,308, 95% CI: 24.02%−24.18%), respectively. The number of post views, reposts, and adds to favorites decreased simultaneously after peaking on the day of posting. Figure 2 shows the engagement of followers 7 days after posting. Table 1 reveals the characteristics about the engagement of followers to posts by the Guangzhou I-Health OWA. For post views, the highest median was chronic disease posts, and the highest maximum was infectious disease posts. Regarding the number of reposts, the highest median was child and adolescent health posts, while the highest maximum was infectious disease posts. About the number of users adding to favorites, the highest median was environmental health posts, while the highest maximum was vaccine-related event posts.

**Table 1:** Engagement of followers for the posts posted by the Guangzhou CDC I-Health official WeChat account.

**Variables**	***n* (%)**	**Post views**					**Share**					**Add to favorites**				
		**Percentile**			**Min**.	**Max**.	**Percentile**			**Min**.	**Max**.	**Percentile**			**Min**.	**Max**.
		**25**	**50**	**75**			**25**	**50**	**75**			**25**	**50**	**75**		
**TYPE OF POSTS**																
Organizational promotion	50 (23.47)	634.5	1203.5	2656.5	314	64,881	15.5	61.5	161.25	0	7,540	0	3	9.5	0	176
Infectious disease	61 (29.64)	2,150	4,054	7,449	803	98,823	83	169	359	11	11,400	3	7	27	0	245
Vaccine-associated event	21 (9.86)	1325.5	3,946	7596.5	452	33,852	70.5	179	477	6	4,782	6	12	56.5	0	834
Environment health	16 (7.51)	1844.75	3,491	5279.75	891	33,722	101.75	220.5	370	41	1,546	10.25	21.5	36.75	2	98
Nutrition and food-borne disease	35 (16.43)	2892.25	4,229	6317.25	1,356	14,537	121.25	242	377.5	39	897	5.25	11	21.75	2	54
Chronic disease	25 (11.74)	2,797	4,733	6,221	729	11,530	121	216	435.5	32	826	5.5	16	37	0	114
Child and adolescent health	5 (2.35)	3001.5	3,790	8878.5	2,672	13,666	132	247	706.5	71	1,049	7.5	13	104.5	6	195
**CHARACTERISTIC OF POSTS**																
Original post	195 (91.55)	2,013	3,929	6,389	314	98,823	79	182	385	0	11,400	3	11	26	0	834
Repost	18 (8.45)	893	1119.5	1842.75	452	4,036	38.25	74	96.25	3	557	0.75	3.5	8.25	0	51
**EFFECTIVENES**																
Focused on hot spots	48 (22.54)	2434.75	3,929	7159.75	836	64,881	126.75	220	600.5	20	7,540	6	11	25.25	1	313
Common event	165 (77.46)	1504.5	3,306	5957.5	314	98,823	58.5	155	349	0	11,400	2	9	23	0	834
**HEADLINE**																
Yes	186 (87.32)	2356.25	4,040.5	6,560	475	98,823	96.75	191	397.5	3	11,400	5	11	27	0	834
No	27 (12.68)	502	729	912	314	1,804	7	18	42	0	557	0	0	4	0	67

Table 2 reveals the factors associated with the engagement of followers for posts published by the GZCDC i-Health OWA. The multiple logistic regression model showed that the number of post views was positively correlated to infectious disease posts (AOR: 3.20 95% CI: 1.16–8.81), original posts (AOR: 10.20, 95% CI: 1.17–89.28), and posts where the title reflected the content (AOR: 2.93, 95% CI: 1.16–8.81). In addition, the number of reposts was positively associated with nutrition and food-borne disease posts (AOR: 3.57, 95% CI: 1.24–10.27), chronic disease posts (AOR: 3.26, 95% CI: 1.03–10.31), headline posts (AOR: 5.01, 95% CI = 1.36–18.51), and posts focused on current events (AOR: 2.11, 95% CI = 1.02–4.47). The number of adding to favorites had a positive correlation with chronic disease posts (AOR: 4.30, 95% CI: 1.31–14.13), headline posts (AOR: 6.19, 95% CI = 1.62–23.58), and posts where the title reflected the content (AOR: 2.43, 95% CI: 1.12–5.28).

**Table 2:** Factors associated with engagement of followers on posts by the Guangzhou CDC I-Health official WeChat account.

**Independent variables**	**N (%)**	**Association with engagement of followers [aOR(95% CI)]**		
		**Post views**	**Share**	**Add to favorites**
**TYPE OF POSTS**				
Organizational promotion	50 (23.47)	Ref	Ref	Ref
Infectious disease	61 (29.64)	3.20 (1.16, 8.81)^*^	1.97 (0.78, 4.98)	1.43 (0.57, 3.60)
Vaccine-associated event	21 (9.86)	2.68 (0.72, 10.02)	2.74 (0.80, 9.38)	3.24 (0.93, 11.29)
Environment health	16 (7.51)	1.33 (0.36, 5.00)	2.13 (0.59, 7.66)	6.24 (1.40, 27.82)^*^
Nutrition and food-borne disease	35 (16.43)	2.60 (0.88, 7.70)	3.57 (1.24, 10.27)^*^	3.34 (1.17, 9.59)^*^
Chronic disease	25 (11.74)	3.18 (0.94, 10.76)	3.26 (1.03, 10.31)^*^	4.30 (1.31, 14.13)^*^
Child and adolescent health	5 (2.35)	3.42 (0.44, 26.61)	7.76 (0.72, 83.62)	3.18 (0.42, 23.90)
**CHARACTERISTIC OF POSTS**				
Original post	195 (91.55)	10.20 (1.17, 89.28)^*^	4.94 (0.99, 24.65)	5.77 (1.14, 29.19)
Repost	18 (8.45)	Ref	Ref	Ref
**EFFECTIVENESS**				
Focused on hot spots	48 (22.54)	0.81 (0.39, 1.69)	2.11 (1.02, 4.47)^*^	0.96 (0.46, 2.00)
Common event	165 (77.46)	Ref	Ref	Ref
**HEADLINE**				
Yes	186 (87.32)	1.22 (0.53, 5.32)	5.01 (1.36, 18.51)^*^	6.19 (1.62, 23.58)^**^
No	27 (12.68)	Ref	Ref	Ref
**THE TITLE REFLECTS THE CONTENT**				
Yes	164 (77.00)	2.93 (1.16, 8.81)^*^	1.43(0.67, 3.03)	2.43 (1.12, 5.28)^*^
No	49 (23.00)	Ref	Ref	Ref

*AOR, adjusted odds ratio*.

* *p < 0.05*,

** *p < 0.01*.

*The bold values meant that they are statistically significant. In other words, the P value is less than 0.05, 0.01 or 0.001*.

## Discussion

To the best of our knowledge, this is the first study by the CDC to explore the influencing factors on followers' engagement of the OWA of the CDC in Guangzhou, South China. Our study showed that the accumulated number of followers of the GZCDC i-Health OWA increased steadily, which indicated the potential application perspective of the OWA to enhance health information and public health literacy. The volume of post views and shares peaked on the first day of the post, and dropped sharply to the very low level in the following 7 days. The key period for health information and dissemination of health literacy was the first 7 days. Although we did not take the week day and hours of posts into consideration, a prior study showed that most Facebook posts were posted during weekends, and the majority of posts per day fell between midnight and early morning (14). The finding reminded us that posts of the OWA should be posted during this time period to ensure better dissemination and even use certain measures to encourage the public to share the posts, such as a lucky draw with a gift reward. Furthermore, we could use the data of posts to facilitate our infectious disease surveillance. A model study indicated that 1 week-lagged Zika tweets were best correlated with weekly ZIKV cases (27). The application of public account monitoring in the early warning and prediction of infectious diseases should be strengthened in the future.

The median of the top three post views was chronic disease, nutrition and food-borne disease, and infectious disease, which indicated that these topics were more appealing to readers. This finding was almost the same as the prior study, which revealed that articles about infectious disease, food safety and nutrition, vaccination, and health life style were inclined to get more post views (28). It hinted that these kinds of issues should be strengthened in the OWA health information in future. We found that most followers of the OWA were women, which was different from other social media. For example, Facebook and Instagram have a relative balance of male and female users, while Twitter gets more male users. It may be attributed to the fact that women were more interested in gaining health information than men. Women are known to be some of the most prolific social media users (1). This may also contribute to the higher rates of engagement (29, 30). Meanwhile, we should find ways to attract

more male readers to expand the influence and effectiveness of health information.

Our analysis showed several characteristics of posts that were positively associated with health information dissemination. The headline was positively associated with the amount of shares, and adds to favorites. A recent study during the COVID-19 outbreak in China manifested that the headline played a positive role in a post's popularity (31). It reminded us that we should use the headline to transmit the most important and even emergency health information for better dissemination.

Social media engagement is one of the indicators for the effectiveness of health information (32). A study focused on Facebook revealed strong associations between health education posts and risk communication posts with good engagement rates (14). Different types of posts have different features that affect the engagement of the followers of an OWA. The content of articles was correlated to the users' engagement and was identified as an essential factor to determine whether WeChat users forward or share articles with friends (33). Studies of other social media platforms have also shown that the content of posts appears to have a significant effect on user engagement (22). In our study, people tended to share chronic disease and nutrition and food-borne disease posts, while infectious diseases posts had a more positive association with post views than organizational promotion posts. Original posts contributed to higher post views, which reminds us to use original posts in health information as much as possible. Adding to favorites meant that the article was useful to the readership, which is positively associated with the headline, title-reflected content, and type of posts with chronic disease. Organizational promotion was set as the reference in our research, and some studies pointed out that many health organizations were still focused on “pushing” organizational promotion posts to users, rather than encouraging participation and engagement-related content on social media (34, 35). A previous study showed that organizational promotion posts were correlated to low engagement rates. This phenomenon could be attributed to the fact that such posts focus on organization interest more than public interest (36). The features mentioned above suggested that we should choose an appropriate combination to improve the effectiveness of health information dissemination. Meanwhile, it is important to pay attention to methods of delivering messages. Multimedia applications, such as graphics, video, or pictures, make them more accessible to the public when understanding health information (31). However, the engagement was not the same as actual health-related behavior, and the relation between them is still to be investigated in the future.

In addition, the current results revealed that popular science articles were also popular with users, such as an article entitled “Running hot spots.” Our study showed that people liked to share posts with hot spots to their moments and friends, however it did not result in or contribute to higher amounts of post views and adds to favorites. A previous study demonstrated that articles with just hot spots were less likely to obtain high-level reading and liking than those with none (28). In other words, it implied that posts with hot spots should still be combined with high quality content (31), headlines, and other marketing elements (28) to achieve a better propagation effect. We should conduct secondary dissemination of health information with propaganda efforts, such as efforts related to major livelihood projects, making use of the advantages of traditional media with a solid audience base, to gain wider attention (37–39).

Our study has certain limitations. First, it was a cross-sectional study, so it could not obtain the causal correlation. Second, our results were from the OWA of the Guangzhou Center for Disease Control and Prevention, so it could not represent that of other provinces or cities, and our sample size was limited, which will lead to a wide confidence interval. Third, it was not possible for us to eliminate the likelihood that some potential confounding factors existed in our research, including demographic factors, communication skills, geographical factors, the status of the epidemic, and preventive measures, moreover the effect of number of figures on health information was not taken into consideration in our study.

## Conclusions

In the current study, we conducted an analysis of the official GZCDC WeChat account, including its post content, composition of followers, and engagement of followers, to understand reading preferences, habits, and associated factors. We should seize the first 7 days for health information dissemination and try to attract more male readers to expand the influence and effectiveness of health information. Meanwhile, we could take advantage of the headline to transmit the most important health information for better dissemination, especially combined with hot spots content.

Our findings elaborated the current status of the OWA of the GZCDC, and facilitate government departments to formulate better strategies and improve the effectiveness for public health information dissemination. Moreover, our results of the CDC's WeChat study could provide a hint for health information launched in other social media platforms around the world, such as Facebook, Instagram, and Twitter.

## Data Availability Statement

The raw data supporting the conclusions of this article will be made available by the authors, without undue reservation.

## Author Contributions

WL: conceptualization and writing—review and editing. XM and JL: data curation, methodology, and writing—original draft. XM and WL: supervision and validation. JL: visualization. All authors contributed to the article and approved the submitted version.

## Conflict of Interest

The authors declare that the research was conducted in the absence of any commercial or financial relationships that could be construed as a potential conflict of interest.

## Publisher's Note

All claims expressed in this article are solely those of the authors and do not necessarily represent those of their affiliated organizations, or those of the publisher, the editors and the reviewers. Any product that may be evaluated in this article, or claim that may be made by its manufacturer, is not guaranteed or endorsed by the publisher.

## Abbreviations

OWA: official WeChat account
CDC: disease control and prevention
GZCDC: Guangzhou Center for Disease Control and Prevention
GZCDC i-Health OWA: Guangzhou Center for Disease Control and Prevention i-Health official WeChat account
AOR: adjusted odds ratio
95% CI: 95% confident interval.
